# Development and Mechanical Characterization of Artificial Skin for Surgical Suture Training: Tensile Strength and Viscoelastic Properties Compared to Human Skin

**DOI:** 10.7759/cureus.81123

**Published:** 2025-03-24

**Authors:** Todor G Bogdanov, Todor A Hikov, Krasimir K Yanev, Konstantinos E Papadakis, Rene D Mileva-Popova

**Affiliations:** 1 Medical Physics and Biophysics, Medical University - Sofia, Sofia, BGR; 2 Dermatology and Venerology, Medical University - Sofia, Sofia, BGR; 3 Physiology and Pathophysiology, Medical University - Sofia, Sofia, BGR

**Keywords:** artificial skin, educational model, surgical suturing training, tensile strength, viscoelastic properties

## Abstract

The effective training of medical students in surgical suturing techniques requires realistic and accessible artificial skin models. This study presents the design, fabrication, and mechanical characterization of a low-cost, 3D-printable artificial skin model that replicates the viscoelastic and tensile properties of human skin. The model is designed to be scalable, with working areas ranging from 100 cm² (for single suture practice) to 10,000 cm² (for large-scale simulations).

The artificial skin consists of two 1-mm-thick layers, simulating the epidermis and subcutaneous fat, supported by a porous sponge base housed in a 3D-printed adjustable holder. The model was fabricated using readily available, inexpensive materials to ensure cost-effectiveness and accessibility. Tensile tests were performed using a 3-0 monofilament polypropylene suture, measuring the force required to rupture the artificial skin under uniaxial tension. Additionally, viscoelastic properties were evaluated through standard procedure for Young's modulus determination.

Results indicate that the mechanical behavior of the artificial skin closely matches the tensile strength and viscoelastic properties of human skin. The model provides a realistic and adaptable platform for medical training while remaining affordable and easy to produce. Future work will focus on optimizing the material composition and refining the skin structure for improved biomechanical accuracy and durability in surgical education.

## Introduction

Surgical suturing is a fundamental skill that medical students must develop early in their training. However, access to real patients for hands-on practice is highly restricted or even impossible at early stages of medical education due to ethical and safety concerns. The availability of realistic and cost-effective surgical suturing models is essential for medical training. Previous studies have explored simplified suturing models for preclinical training [1] and investigated how repetitive mechanical stress affects porcine skin [2], providing valuable insights for the development of improved training models. As a result, students rely on artificial models and alternative materials to develop their suturing techniques before transitioning to real clinical scenarios. The ability to practice repeatedly in a controlled environment is crucial for mastering different suture techniques, understanding tissue handling, and improving hand dexterity. Without sufficient training, students may struggle with essential aspects of wound closure, such as knot security, tension control, and wound edge approximation, which are critical for proper healing and minimizing complications.

Recent advancements in synthetic skin technologies have focused on improving material realism and tactile feedback, with some models incorporating multilayered silicone structures or composite materials to mimic the mechanical behavior of human skin better. However, these innovations often come at a high cost or require specialized equipment, limiting their widespread adoption among medical students. Commercially available suture training kits, while significantly more affordable today than in previous years, remain relatively expensive, limiting accessibility for many students. These kits often contain pre-made silicone pads that simulate human skin to some extent, but their material properties may not adequately replicate the viscoelastic behavior and mechanical resistance of real human tissue. Furthermore, many of these training kits have limited reusability, as repeated suturing degrades their structural integrity. While institutions may provide shared practice resources, access to these is often limited, preventing students from engaging in frequent, self-directed practice outside of formal training sessions.

Another widely used approach is practicing on animal tissues, such as pig ears or legs, which are commonly used in medical training due to their availability and relatively low cost. However, these tissues do not accurately mimic human skin's mechanical properties, as they differ in thickness, elasticity, collagen structure, and response to needle penetration. Additionally, animal tissue degrades quickly, requires proper refrigeration, and poses ethical and hygiene-related concerns. Moreover, the use of animal models is not always practical for home practice, as students may lack access to proper facilities for handling biological specimens safely.

To address these challenges, we propose the development of an affordable, accessible, and easy-to-manufacture artificial skin model that students can produce themselves using readily available materials. This model is designed to replicate the mechanical properties of human skin, allowing students to practice different suturing techniques in a realistic setting. Unlike commercial kits, our model offers customizability, as its size and structural properties can be adjusted to accommodate different training needs. Additionally, the use of 3D-printed holders allows for scalability, enabling students to create models ranging from small 100 cm² pads for individual sutures to large 10,000 cm² panels for advanced multi-suture training.

In this study, we evaluate the viscoelastic properties of the artificial skin model and compare them with those of human skin. Additionally, we measure the tensile strength at failure, using monofilament 3-0 polypropylene sutures, and compare it with published data on human skin mechanics. Our goal is to provide a cost-effective, scalable, and reproducible training model that enhances surgical education and improves suturing proficiency among medical students. By ensuring that the model is inexpensive and easy to produce, we aim to democratize access to high-quality surgical training tools, allowing students to refine their skills effectively and confidently before transitioning to real clinical practice.

## Technical report

The developed suturing training model for student training is based on a combination of a silicon rubber compound mix, silicone gel, and the Silc Pig® silicone rubber color system (Ecoflex^TM^, Smooth-On, Inc., Macungie, PA), combined with a four-way stretch fabric, a porous sponge, and a 3D-printed frame (using PLA filament 3Dline (3D Line, Yambol, Bulgaria)), printed on a Bambu Lab X1 Carbon 3D Printer (Bambu Lab, Austin, TX). The frame design was created using Shapr3D (Shapr3D Zrt., Budapest, Hungary).

The original idea for creating artificial skin for surgical training was inspired by the YouTube channel of Smooth-On, which contains multiple videos on artificial skin creation. However, the challenge of realism and applicability in medical student training arose, prompting the development of a more suitable model.

**Figure 1:**
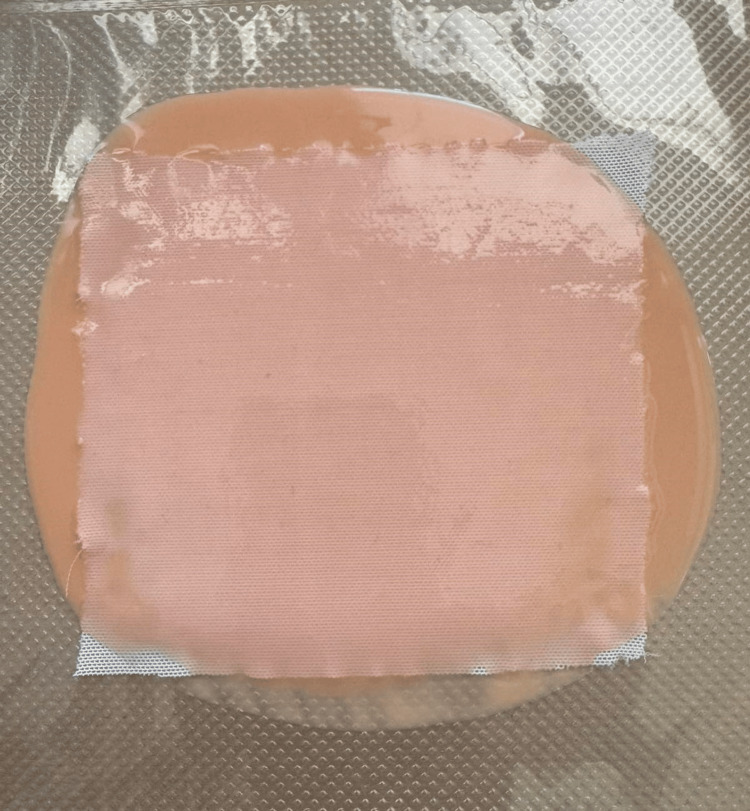
Suturing training model at stage 1 of the preparation

To manufacture the model, a two-component silicon rubber compound 00-30 was used to create the skin-like surface, and silicone gel was employed to simulate subcutaneous adipose tissue. The artificial skin model was fabricated using a combination of silicone rubber, silicone gel, and 3D-printed structural elements. Previous studies have demonstrated the applicability of 3D printing in surgical simulations [3] and the importance of using tissue phantoms to study the mechanical properties of injured and sutured skin [4]. A 1:1 ratio of component A and component B of the silicon rubber compound, with an added pigment, was mixed on a pre-prepared horizontal surface covered with a nylon material to minimize adhesion. The mixing process involved adding the pigment to component B first, followed by homogenization for two minutes (Figure 1). Then, component A was introduced, and the mixture was stirred for another two minutes. After allowing the mixture to activate for five minutes, it was poured onto the mold. The manufacturer reported that the working time is 15 minutes; however, our experiments showed that it could extend up to 20 minutes at 20°C and to 48%-55% relative humidity. Immediately after pouring, a 12×12 cm piece of the four-way stretch fabric was placed on top, allowing absorption due to surface tension and the wetting properties of the fabric. This addition enhances mechanical strength while preserving elasticity. The manufacturer specifies a cure time of two hours.

After this period, a silicone gel mixture (1:1 ratio of components A and B) was poured over the model with a total weight of 30 grams. Pigment could be optionally added. The mixing procedure was identical to that of the silicon rubber compounds. The working time for silicone gel is significantly longer, 45 minutes according to the manufacturer and up to 60 minutes in our observations. The cure time is four hours, but we recommend allowing 20 hours before separating the training model from the nylon sheet to ensure complete curing. The given material amounts allowed for layers of 1 mm thickness per simulated tissue type over an 18×18 cm area. If poured into a pre-printed holder with defined dimensions, height markers should be placed inside to control the pouring height. It is important to note that greater layer thickness increases cure time.

**Figure 2:**
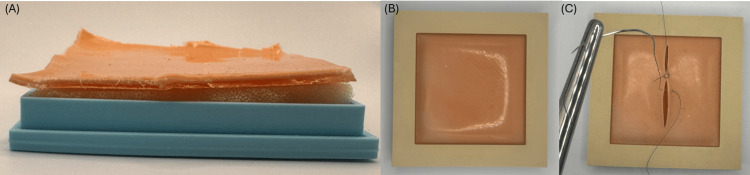
Model structure (A), finished one (B), and cut one (C)

Following the curing process, a 78×78 mm section was cut from the model (Figure 2A). A porous sponge of the same size and 12 mm thickness was placed inside a 3D-printed frame with internal dimensions of 80×80 mm. The suturing training model was positioned on top, and the structure was closed with a cover overlapping by 10 mm on all sides, leaving a 60×60 mm working opening (Figure 2B and Figure 2C).

The developed suturing training model was assessed in terms of cost-effectiveness and realism. While its production cost is significantly lower than commercial models, its key feature is its accurate representation of human skin properties. To validate this, mechanical strength and elasticity tests were conducted. The cost of the model depends on the current prices of the materials used. At the time of its creation, EcoFlex® 00-30 and EcoFlex® Gel were available on online marketplaces at approximately 100 USD for 900 grams of each. The four-way-stretch fabric and porous material could be found at around 5 USD per square meter, while PLA filament was priced at about 15 USD per kilogram. To produce a 100 cm² (10×10 cm) model, approximately 14 grams of EcoFlex 00-30, 14 grams of EcoFlex Gel, and 20 grams of PLA filament were used. Based on these values, the production cost of a single model amounts to less than 2 USD. Commercially available surgical suture training models vary widely in price, ranging from $15 for basic silicone pads to over $500 for high-fidelity anatomical simulators. Compared to these, the presented model offers a highly affordable alternative with a production cost of under $2.

Using an HLB Test Stand+HP-500N (Mxmoonfree, China), we measured the tensile force applied to a Non-Absorbable Surgical Suture U.S.P. 3-0 monofilament (Meril Endo Surgery Private Limited, Gujarat, India) required to tear the suturing training model at various distances from the incision edge. The results are presented in Table 1.

**Table 1:** Results from tensile tests

Distance (mm)	Average force (N)	Test 1 (N)	Test 2 (N)	Test 3 (N)	Test 4 (N)	Test 5 (N)	Test 6 (N)	Test 7 (N)	Test 8 (N)	Test 9 (N)	Test 10 (N)
1	86.11	85.6	87.5	86.4	83.1	85.5	86.3	87.3	86,9	87.4	85.9
3	100.48	100.1	101.4	100.8	98.5	102.3	100.9	99.8	98.5	102.2	100.3
5	120.43	121.4	120.6	120.7	120.2	119.8	122.1	120.9	120.6	119.1	118.9
7	136.79	135.4	136.3	134.3	142.5	137.3	143.4	131.1	140.2	137.2	130.2

**Figure 3:**
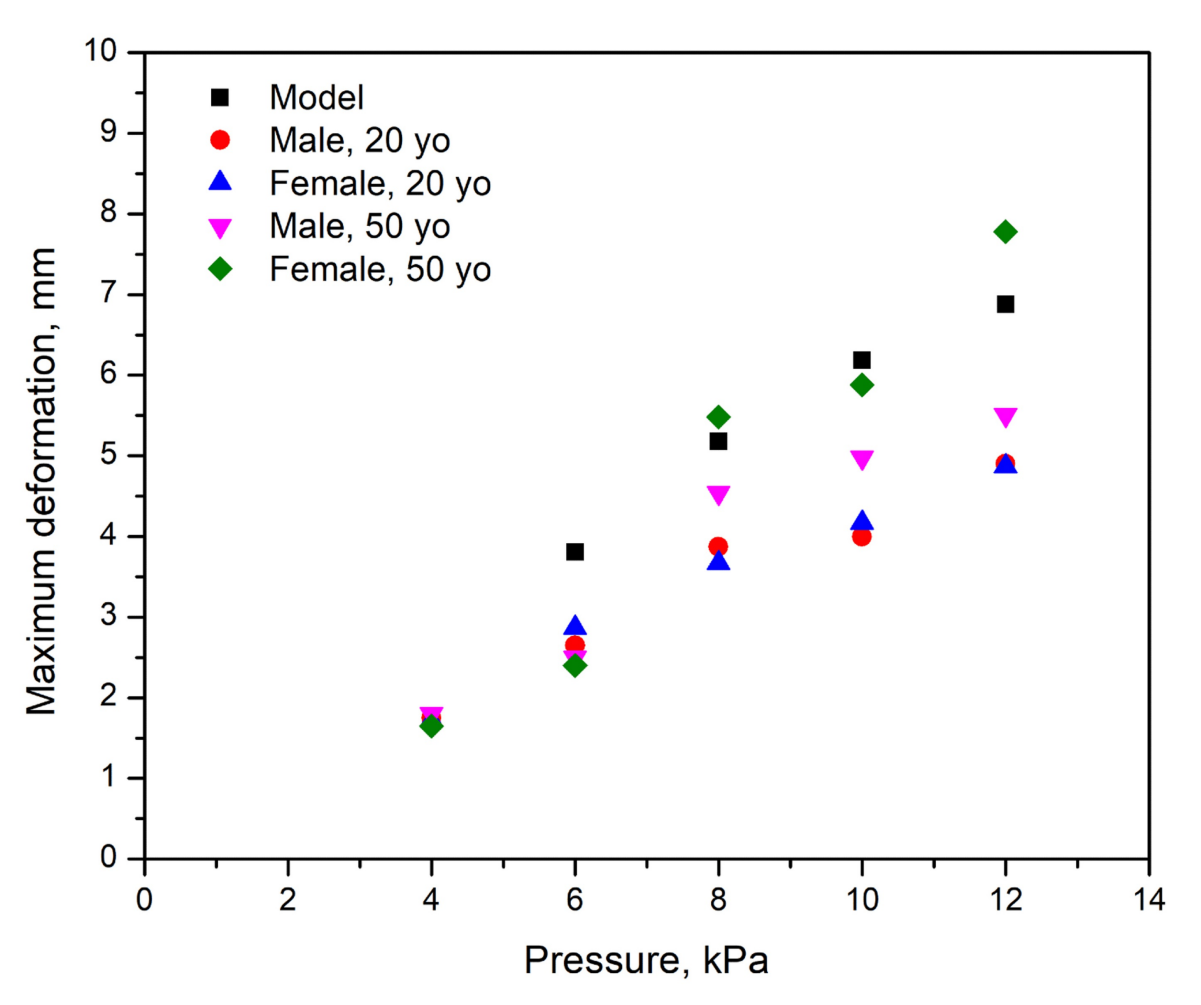
Maximum deformation depending on applied suck pressure for the model (black dots), 20-year-old male (red dots), 20-year-old female (blue triangles), 50-year-old male (purple triangles), and 50-year-old female (green squares)

A suction test was conducted to determine the viscoelastic properties of the presented model, specifically its Young's modulus, and compared to human forearm skin in a 20-year-old male, a 20-year-old female, a 50-year-old male, and a 50-year-old female. The maximum deformation as a function of applied suction pressure is shown in Figure 3.

The calculated elasticity moduli are summarized in Table 2.

**Table 2:** Calculated elasticity moduli

	Model	Male, 20 years old	Female, 20 years old	Male, 50 years old	Female, 50 years old
E, kPa	1.56	2.05	2.03	1.87	1.71

A trend of decreasing elasticity with age was observed, with the developed model exhibiting a lower elasticity modulus than the measured values in human subjects.

The developed suturing training model demonstrates realistic mechanical properties for surgical training applications. The cost-effectiveness and high adaptability of this model make it a valuable tool in medical education. Further refinements in material composition and thickness control could enhance the model's resemblance to human skin.

## Discussion

The developed suturing training model was subjected to mechanical testing to assess its strength and elasticity in the context of surgical suturing. The measured tensile strength values at different distances from the incision edge showed a clear trend: as the distance from the wound edge increased, a greater force was required to tear the material. The lowest measured average value was 86.11 N at a 1 mm distance, while the highest was 136.79 N at a 7 mm distance. These data confirm that the mechanical behavior of our model aligns with expectations for real tissues, where deeper-placed sutures provide greater stability.

Comparing our results with published data [5], it is evident that the tensile strength of our model is lower than that of real human skin. In their study, which compared different surgical suturing techniques on human cadaveric skin, the highest measured tear resistance value was 211.13 N for the Lindeque locking suture, while the lowest was 120.79 N for the horizontal mattress suture. These values indicate that while our model provides an opportunity for realistic mechanical testing of surgical sutures, its mechanical strength remains below that of real skin. This discrepancy may be attributed to the materials used, particularly the properties of silicone rubber compound 00-30, which, although offering some elasticity, does not fully replicate the structure of human dermis.

Our tensile strength measurements indicate that the artificial skin model exhibits lower mechanical resistance than real human skin. Similar studies on rat skin [6] and porcine mitral valve chordae tendineae [7] suggest that variations in tissue composition and elasticity significantly impact tensile performance, which should be considered in future refinements of artificial skin models. Another possible explanation for the differences could be the testing methodology. In our measurements, the tensile force was applied to the silicone rubber compound with a single puncture, whereas in the published scientific results, and specifically in the cited study, the tensile force was measured on a completed surgical suture. This distinction is crucial, as a properly placed suture distributes tension more evenly, whereas a direct puncture test focuses stress on a single point, leading to lower recorded values.

Apart from tensile strength, it is important to consider the elasticity of the developed model. Our suction test measurements revealed that the presented model has a lower Young's modulus compared to real human skin. According to published results [8], the Young's modulus of human skin ranges between 5 kPa and 140 MPa, depending on the anatomical location and the measurement methodology. In our case, the recorded values ranged between 1.56 kPa and 2.05 kPa, which is significantly lower than those observed in real human skin. Additionally, a discrepancy exists between the Young's modulus values measured for real human skin in the cited study [8] and those reported by the authors. These differences are likely due to variations in testing methods and/or the absence of the complex fibrous structure of the dermis in our model, which plays a crucial role in the mechanical resilience and elasticity of human skin.

The test results indicate that the model possesses key characteristics that make it suitable for training medical students and surgeons. However, there are opportunities for improvement, particularly in mechanical strength and elasticity.

One possible approach to increasing tensile strength is thickening the layers or using more durable polymeric mixtures. The addition of reinforcing layers of stretchable textile, elastic mesh, or other supportive fabrics could help achieve mechanical properties closer to those of human skin.

To better replicate skin elasticity, biocompatible elastomers with properties similar to collagen and elastin fibers in the dermis could be used. Additionally, a multilayered structure could be implemented, where each layer simulates a specific part of real skin (epidermis, dermis, and hypodermis). However, this approach would increase costs and complexity, making it less practical for an affordable surgical suturing training model.

Although our model exhibits lower mechanical performance compared to real skin, its affordability and low production cost make it an attractive option for training purposes. Commercially available surgical suturing trainers are significantly more expensive and do not always provide adaptability to training needs. In this context, our model represents a flexible and economical alternative that can be modified and improved based on the specific requirements of trainees.

## Conclusions

The developed artificial skin model demonstrates promising potential for use in surgical suturing training due to its cost-effectiveness and adaptability. While the model does not fully replicate the mechanical properties of human skin, it provides a reasonable approximation for practicing various suturing techniques. The comparison with published data highlights some limitations, particularly in tensile strength and elasticity, which may be addressed in future iterations through material optimization and structural refinement.

Further improvements in material composition, such as the introduction of reinforced layers or biocompatible elastomers, could enhance the mechanical accuracy of the model. Despite these limitations, the affordability and ease of production make this model a valuable training tool for medical students and early-career surgeons, providing them with an accessible and reproducible platform to refine their suturing skills before transitioning to clinical practice.
